# Supplementary material for: The Effects of DDI1 on Inducing Differentiation in Ovine Preadipocytes via Oar-miR-432
Source: Int J Mol Sci. 2023 Jul 17;24(14):11567. doi: 10.3390/ijms241411567 (PMC10380388; doi:10.3390/ijms241411567)
Supplement: Supplementary file 1 [file ijms-24-11567-s001.zip › ijms-2475825-supplementary.pdf]

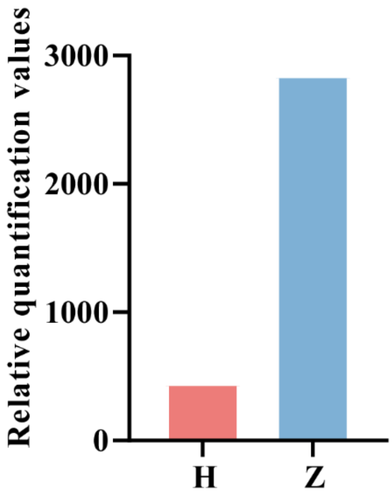

Figure S1: RNA-seq analysis of oar-miR-432 expression in the tail fat tissue of fat-tailed (H) and thin-tailed sheep (Z).

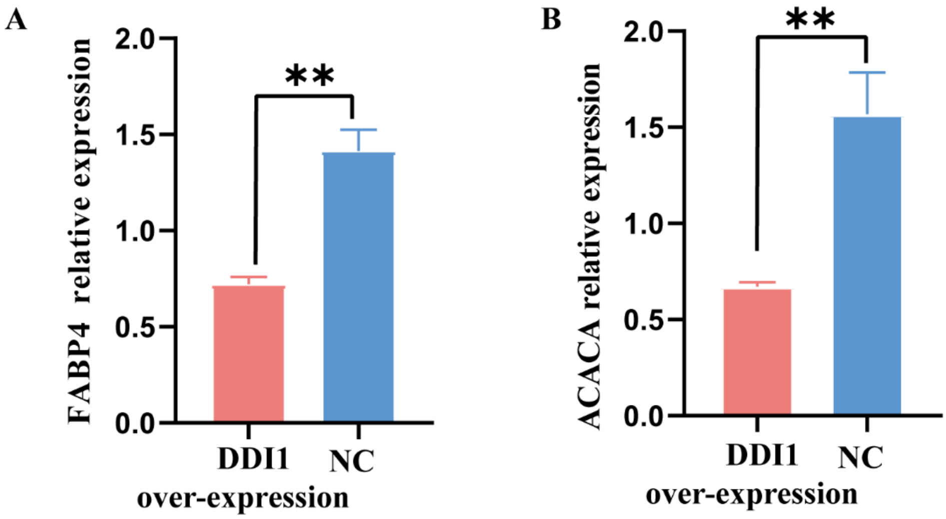

Figure S2: Relative expression of *FABP4* and *ACACA* when the *DDI1* overexpression and their NC were transfected into preadipocytes. Data are presented as mean ± SEM (n = 3). $^{**}P < 0.01$ and $^{*}P < 0.05$.

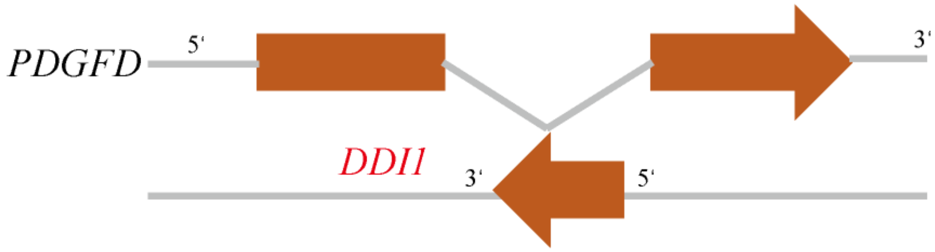

Figure S3: *DDI1* gene sequence was completely located in the intron 1 of *PDGFD* gene. Because the overlapping region is limited to introns for *PDGFD*, there will be no overlapping the mRNAs.

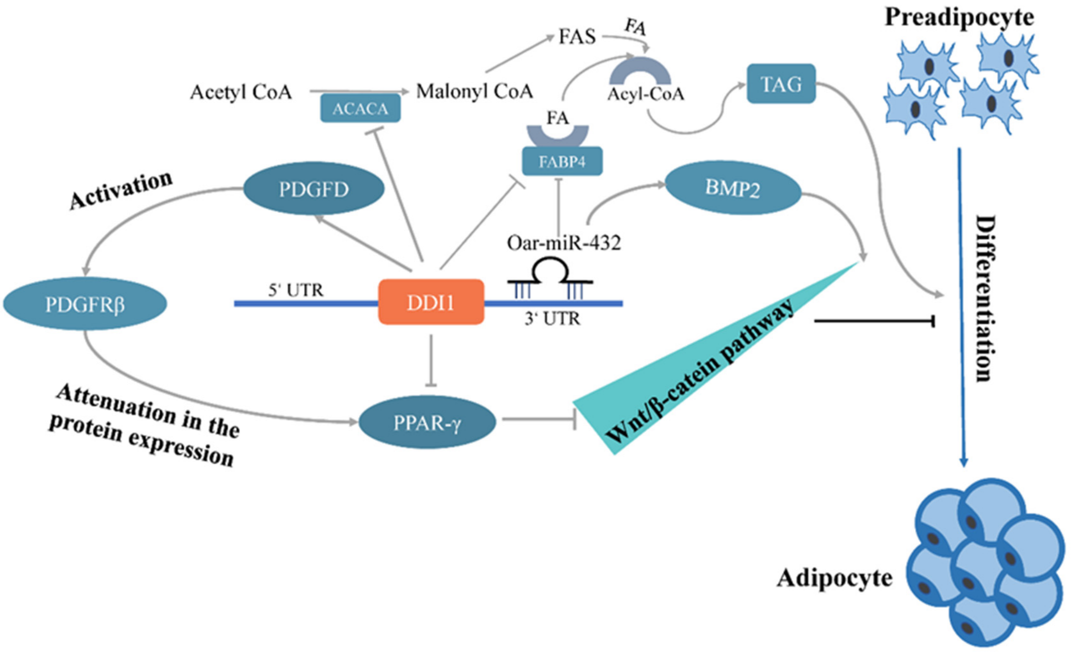

Figure S4: Schematic diagram illustrating how the mechanism of oar-miR-432 mediation by DDI1 modu-lates fat deposition development via the pathway.
